# *Pneumocystis jirovecii* pneumonia in a human caused by long-term use of veterinary drug oclacitinib: A case report

**DOI:** 10.1016/j.idcr.2025.e02459

**Published:** 2025-12-11

**Authors:** Keisuke Oshima, Ryo Koyama, Takashi Akimoto, Toshihiko Nishioki, Tomohito Takeshige, Junko Watanabe, Daisuke Usuda, Kazuhisa Takahashi

**Affiliations:** a Department of Respiratory Medicine, Juntendo University Nerima Hospital, 3-1-10 Takanodai, Tokyo, Nerima, Japan; b Department of Emergency and Critical Care Medicine, Juntendo University Nerima Hospital, 3-1-10 Takanodai, Tokyo, Nerima, Japan; c Department of Respiratory Medicine, Juntendo University Graduate School of Medicine, 3-1-3 Hongo, Tokyo, Bunkyo, Japan

**Keywords:** *Pneumocystis jirovecii*, *Pneumocystis* pneumonia, Oclacitinib, JAK inhibitor, Veterinary drug

## Abstract

*Pneumocystis jirovecii* pneumonia (PJP), an opportunistic fungal infection, typically occurs in immunocompromised patients, such as those with human immunodeficiency virus (HIV) infection or those receiving prolonged immunosuppressive therapy. Recently, PJP in non-HIV patients treated with novel immunomodulatory agents, including Janus kinase (JAK) inhibitors, has been increasingly reported. Here, we report a case of PJP in a 53-year-old HIV-negative Japanese man with no recognized immunosuppressive comorbidities. The patient, a veterinarian, had been self-administering oclacitinib (16–64 mg/day), a selective JAK1 inhibitor approved for the treatment of atopic dermatitis in dogs, daily for approximately 2 years to manage atopic dermatitis. He presented with progressive exertional dyspnea and fever. Chest computed tomography revealed bilateral ground-glass opacities with patchy consolidations. The diagnosis of PJP was confirmed by polymerase chain reaction of bronchoalveolar lavage fluid and Grocott’s methenamine silver staining of transbronchial lung biopsy specimens. He was initially treated with trimethoprim-sulfamethoxazole and corticosteroids; however, the regimen was switched to atovaquone owing to hepatotoxicity. The patient recovered fully and remained recurrence-free at 1-year follow-up. No other causes of immunosuppression were identified, and oclacitinib use was considered the likely precipitating factor. To our knowledge, this is the first reported case of PJP associated with oclacitinib use in humans. As JAK inhibitors are increasingly being used, clinicians should be aware of their potential to cause opportunistic infections, even with veterinary formulations without approved human indications.

## Highlights

• First known case of oclacitinib-associated *Pneumocystis jirovecii* pneumonia (PJP).
• Oclacitinib is a Janus kinase inhibitor approved for veterinary, but not human use.
• 53-year-old HIV-negative Japanese man presented with exertional dyspnea and fever.
• Diagnosed using PCR (bronchoalveolar lavage) and GMS staining (lung biopsy specimen).
• Treatment: trimethoprim-sulfamethoxazole, corticosteroids, followed by atovaquone.

## 1 Introduction

*Pneumocystis jirovecii* pneumonia (PJP) is an opportunistic fungal infection that primarily affects individuals with impaired cell-mediated immunity, such as patients with human immunodeficiency virus (HIV) infection, hematologic malignancies, or prolonged immunosuppressive therapy. Although the incidence of HIV-associated PJP has declined with the advent of antiretroviral therapy, non-HIV-associated cases in immunocompromised populations have been increasing, particularly among patients receiving novel immunomodulatory agents, including biologics and kinase inhibitors [1].

Among these, Janus kinase (JAK) inhibitors have been recognized as a potential risk factor for opportunistic infections [2]. JAKs are intracellular tyrosine kinases involved in immune regulation, and their inhibition can impair the host defense against pathogens [3]. JAK inhibitors are approved for the treatment of various diseases, including rheumatoid arthritis, atopic dermatitis, ulcerative colitis, and myeloproliferative disorders [4], [5]. Herpes zoster is the most frequently reported opportunistic infection associated with JAK inhibitor therapy; however, cases of PJP have also been documented, although less commonly [6].

Oclacitinib is a selective JAK1 inhibitor approved for the treatment of atopic dermatitis in dogs aged 12 months or older [7]. Despite the absence of approval for human use, it can be obtained in Japan through online sources. To date, only one report on the human use of oclacitinib has been published, in which the drug was administered for 6 months for atopic dermatitis, and no adverse events were noted [8]. Additionally, PJP has not been reported in association with the veterinary use of oclacitinib in dogs. To the best of our knowledge, no cases of PJP associated with the oral use of veterinary JAK inhibitors have been reported.

Here, we report the first case of PJP in an HIV-negative patient without recognized immunosuppressive comorbidities, in whom long-term self-administration of oclacitinib was considered the likely precipitating factor.

## 2 Case presentation

**Fig. 1:**
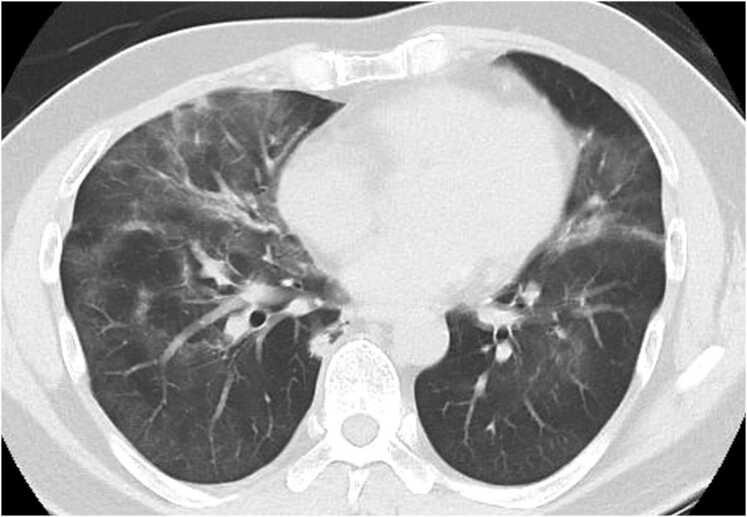
Chest computed tomography images obtained before the initiation of treatment. A plain chest CT scan obtained before treatment initiation shows diffuse, map-like ground-glass opacities throughout both lungs, with a predominance in the middle and lower lobes.

A 53-year-old Japanese man presented to a local clinic with a persistent cough lasting more than 1 month and exertional dyspnea of 2 weeks’ duration. Based on his symptoms, he was diagnosed with bacterial pneumonia, and combination therapy with ampicillin (2250 mg/day)/clavulanate (375 mg/day) and azithromycin (500 mg/day) was initiated. Despite treatment, he developed fever a few days later and his dyspnea continued to worsen. At 1-week follow-up, his oxygen saturation on room air had dropped to approximately 90 %. Chest computed tomography (CT) revealed bilateral ground-glass opacities with a map-like distribution and patchy consolidations, predominantly in the lower lobes (Fig. 1). Based on these findings, atypical or interstitial pneumonia was considered. The laboratory test results are summarized in Table 1. The patient was admitted to the hospital, and his antibiotic regimen was switched to intravenous ceftriaxone (2 g/day). On the following day, serum β-D-glucan was markedly elevated; therefore, oral trimethoprim-sulfamethoxazole (TMP-SMX; TMP 15 mg/kg/day) and voriconazole (400 mg/day) were initiated to cover potential opportunistic infections. Given the need for further evaluation and specialized treatment, the patient was transferred to our hospital 1 week later.

**Table 1:** Laboratory findings before the initiation of treatment.

Parameter (Unit)	Measured Value	Normal Value
White blood cells (10^3^/µL)	7.9	3.3–8.6
Neutrophil (%)	84.0	42–74
Lymphocyte (%)	9.0	18–50
Monocyte (%)	5.0	1–8
Eosinophil (%)	2.0	0–7
Basophil (%)	0	0–2
Hemoglobin (g/dL)	13.7	13.4–17.1
Platelets (10^3^/µL)	300	153–346
Aspartate transaminase (IU/L)	44	13–30
Alanine aminotransferase (IU/L)	44	10–42
Lactic acid dehydrogenase (U/L)	411	124–222
Total bilirubin (mg/dL)	0.7	0.4–1.5
Total protein (g/dL)	6.8	6.6–8.1
Albumin (g/dL)	3.2	4.1–5.1
Creatine kinase (U/L)	162	59–248
Blood urea nitrogen (mg/dL)	11.2	8–20
Creatinine (mg/dL)	0.92	0.6–1
Sodium (mEq/L)	139	135–145
Potassium (mEq/L)	3.8	3.5–5
Chloride (mEq/L)	103	96–107
C-reactive protein (mg/dL)	4.7	0–0.29
Procalcitonin (ng/mL)	0.06	0–0.5
Plasma glucose (mg/dL)	112	65–109
Glycated hemoglobin (NGSP) (%)	6.1	4.6–6.2
Activated partial thromboplastin time (seconds)	37.7	23–38
Prothrombin time (International normalized ratio)	1.02	0.85–1.15
D-dimer (μg/mL)	1.2	0–1

These results were obtained prior to the initiation of treatment at the referring hospital.

He had no history of malignancy, diabetes mellitus, chronic liver disease, chronic kidney disease, autoimmune disease, or prior use of systemic corticosteroids or immunosuppressive agents. A chest CT performed 1 year before this presentation revealed no pre-existing pulmonary abnormalities, indicating the absence of chronic lung disease. Baseline liver and renal function results were within the normal range.

Upon arrival, the combination of markedly elevated β-D-glucan levels and CT findings made PJP the leading diagnostic consideration. Because the patient was a veterinarian with routine exposure to dogs, cats, and birds (including parrots and pigeons), the possibility of zoonotic infections was also considered. Serological testing confirmed negative HIV antibodies, but the cluster of differentiation 4 (CD4) lymphocyte count had decreased to 211/μL. No evidence of aspergillosis, cytomegalovirus pneumonia, or autoimmune diseases was identified. Detailed laboratory findings are summarized in Table 2. CT findings ruled out solid malignancy.

**Table 2:** Serological and microbiological test results.

Parameter (Unit)	Measured Value	Normal Value
Antinuclear antibody	1:20 (speckled)	< 1:40
Anti-SS-A antibody (U/mL)	< 1.0	< 10
Rheumatoid factor (IU/mL)	< 3	≦ 15
Anti-CCP antibody (U/mL)	< 0.6	< 4.5
MPO-ANCA (IU/mL)	< 1.0	< 0.2
PR3-ANCA (IU/mL)	< 1.0	< 0.6
KL-6 (U/mL)	1958	0–500
SP-D (ng/mL)	76.7	0–110
β-D-glucan (pg/mL)	1420	0–20
Aspergillus-specific IgG antibody	Negative	Negative
Aspergillus antigen	Negative	Negative
Cryptococcus antigen	Negative	Negative
Candida antigen	Negative	Negative
IGRA	Negative	Negative
MAC antibody	Negative	Negative
CMV C7-HRP antigenemia assay	Negative	Negative
Anti-HIV antibody	Negative	Negative

These tests, including autoimmune antibody screening and fungal infection markers, were performed at our hospital on day 7 of illness. IGRA: Tuberculosis-specific IFN-γ release assay; MAC: Mycobacterium avium complex; CMV: Cytomegalovirus; HIV: Human immunodeficiency virus

**Fig. 2:**
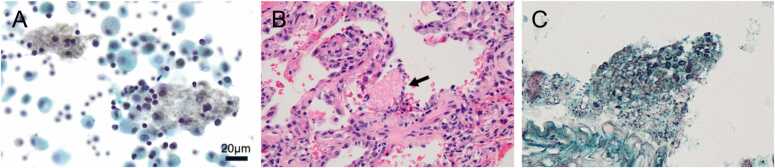
Pathological findings from bronchoscopy. (a) Bronchoalveolar lavage (BAL) was performed from the right B^5^ segment. Cytological examination of the BAL fluid revealed fungal organisms that stained black with Grocott’s methenamine silver, within the alveolar macrophages. (b) Transbronchial lung biopsy was performed from the right B^9^a segment. Hematoxylin and eosin (HE) staining showed eosinophilic, foamy intra-alveolar exudates (arrow). Original magnification, × 200. (c) Grocott’s methenamine silver staining of the lung biopsy specimen demonstrated spherical cysts of *Pneumocystis jirovecii* within the exudates. Original magnification, × 400.

For a definitive diagnosis, bronchoalveolar lavage (BAL) was performed from the right B^5^ bronchus. Cytological examination revealed organisms within alveolar macrophages that stained black with Grocott’s methenamine silver (GMS) stain (Fig. 2a). Polymerase chain reaction analysis of the BAL fluid was positive for *Pneumocystis jirovecii*. Transbronchial lung biopsy (TBLB) was performed from the right B^9^a bronchus. Hematoxylin and eosin (HE) staining demonstrated eosinophilic foamy exudates containing spherical cysts (Fig. 2b), which also stained positively with GMS, consistent with PJP. Based on these findings, the diagnosis of PJP was established.

Detailed history record revealed that the patient had atopic dermatitis and had been self-administering oclacitinib (16–64 mg/day, depending on symptom severity) daily for approximately 2 years. His profession as a veterinarian may have contributed to his decision to self-administer this veterinary JAK1 inhibitor. He reported no use of other prescription or over-the-counter medications, supplements, or health products with immunosuppressive or immunomodulatory effects. He also denied the use of any veterinary medications other than oclacitinib.

**Fig. 3:**
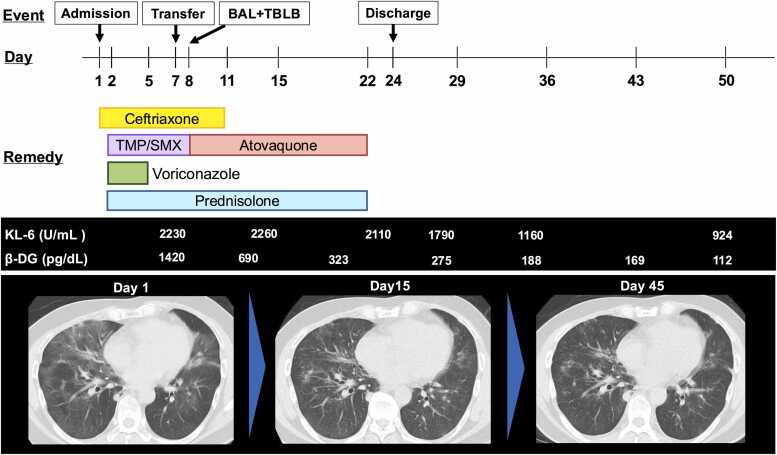
Clinical course of the patient. The patient was admitted to the previous hospital from where treatment was started. On Day 7 of illness, the patient was transferred to our hospital. Based on the pathological findings and other diagnostic results, the diagnosis of PJP was made. Following treatment, his condition improved and he was discharged from our hospital on Day 24. BAL: Broncho-alveolar lavage; TBLB: Transbronchial lung biopsy; TMP/SMX: Trimethoprim and sulfamethoxazole; β-DG: β-D glucan.

At our hospital, TMP-SMX, which had already been initiated at the previous hospital, was continued, together with prednisolone (80 mg/day). On day 7 of therapy, TMP-SMX was switched to atovaquone (1500 mg/day) owing to hepatotoxicity. Following the continuation of therapy, gradual improvement in oxygenation and resolution of bilateral ground-glass opacities were observed. TMP-SMX and atovaquone were administered for a total of 21 days, and prednisolone was tapered and discontinued after 21 days. The clinical course is summarized in Fig. 3.

After completion of PJP treatment, the patient was referred to the dermatology department for management of atopic dermatitis. At 1-year follow-up, no recurrence of PJP was observed.

## 3 Discussion

We present the first known case of PJP in a human with long-term exposure to oclacitinib, where oclacitinib was considered a possible contributing factor. The patient had been self-administering oclacitinib, a selective JAK1 inhibitor approved only for veterinary use. This is an exceptionally rare case of PJP in a non-HIV-infected patient without any known underlying immunosuppressive condition [9]. Reporting this case is important because, to the best of our knowledge, adverse events associated with oclacitinib use in humans have not been previously reported.

In veterinary medicine, oclacitinib has been associated with an increased risk of infections [7], [10], including urinary tract infections and otitis externa, as well as opportunistic infections, such as demodicosis and viral papillomatosis in dogs [10]. Although no cases of *Pneumocystis* pneumonia have been reported in dogs receiving oclacitinib, these findings suggest that the drug may exert immunosuppressive effects under certain conditions.

From a pharmacologic perspective, oclacitinib is a potent and relatively selective JAK1 inhibitor. *In vitro* it inhibits JAK1 at low-nanomolar half-maximal inhibitory concentrations and suppresses multiple JAK1-dependent cytokines, while exerting minimal effects on cytokines that signal through JAK2/TYK2 [11]. Furthermore, human and canine JAK1 share 98 % amino-acid sequence homology, making it highly likely that systemic exposure to oclacitinib in humans would produce a similar JAK1-selective inhibitory profile, even though human pharmacokinetic data are lacking [11].

In humans, JAK inhibitors approved for clinical use, such as tofacitinib and baricitinib, have been linked to an increased risk of serious and opportunistic infections [2], [12]. Regarding the association between JAK inhibitors and PJP, no significant relationship has been demonstrated in large-scale epidemiological studies to date [13]. Nevertheless, given the well-documented association between JAK inhibitors and opportunistic infections and based on several case reports and a few observational studies, a potential association between JAK inhibitor use and PJP has been suggested [6], [14], [15], [16]. These reports include sporadic cases of PJP in patients receiving selective JAK1 inhibitors such as upadacitinib, typically in the presence of additional immunosuppressive factors [16]. Although such data are limited, JAK1 inhibition should be considered one of several potential contributors to PJP in susceptible patients.

In contrast, a recent multicenter retrospective cohort study of patients with rheumatoid arthritis showed that prior use of biologics did not increase the severity or mortality of PJP compared with therapy without biologics [17]. These findings suggest that immunomodulatory agents such as biologics and possibly JAK inhibitors may primarily increase susceptibility to PJP but not worsen the clinical course once infection has developed, although data specific to JAK inhibitors remain limited.

The JAK-STAT pathway plays a central role in mediating immune responses through cytokines such as IFN-γ and GM-CSF [4]. Inhibition of this pathway may impair host defense mechanisms, particularly cell-mediated immunity, thereby increasing the susceptibility to opportunistic infections such as PJP [4].

In the present case, the patient had no history of immunosuppressive diseases or medications, other than the prolonged use of oclacitinib. His occupation as a veterinarian likely facilitated access to this veterinary drug and may have influenced his decision to self-administer it for atopic dermatitis. This case highlights important ethical and social concerns related to self-medication with potent immunomodulatory agents that are not approved for human use. When patients perceive such products as convenient or accessible alternatives to specialist care, they may expose themselves to unrecognized risks and delay appropriate diagnosis and evidence-based treatment. Clinicians and regulatory bodies should recognize these challenges and clearly communicate the dangers of unsupervised use of veterinary or unapproved drugs in humans. Additionally, steps should be taken to ensure adequate access to safe, supervised therapies for chronic conditions, such as atopic dermatitis.

Recent evidence suggests that low-dose TMP-SMX provides efficacy comparable to conventional-dose TMP-SMX in non-HIV PJP, while causing fewer adverse events [18]. In a multicenter study, Nagai et al. reported low-dose regimens provided outcomes similar to conventional dose regimen but with reduced treatment-limiting toxicities [18]. Although we initially used conventional-dose TMP-SMX according to standard practice, therapy had to be discontinued because of hepatotoxicity. These findings indicate that low-dose TMP-SMX may be a reasonable option for patients at risk of intolerance or adverse reactions.

This case report provides several important clinical insights. First, it highlights the importance of thorough history taking. The use of unapproved or veterinary medications may carry unexpected risks, and patients may hesitate to disclose such information to medical staff. Therefore, clinicians must ask targeted questions with a degree of suspicion. Second, although JAK inhibitors are generally considered safe, they can cause serious opportunistic infections under certain conditions. Third, as the clinical use of JAK inhibitors continues to expand, healthcare providers must maintain awareness of rare but potentially serious infections, such as PJP.

This case report had a few limitations. As this report is based on a single patient, the causality between oclacitinib use and the development of PJP cannot be definitively established. Although oclacitinib was the only identifiable potential immunosuppressive factor, the absence of controlled conditions or broader epidemiological data limit the generalizability of our findings. In addition, although microbiological examinations, including staining methods, are helpful for diagnosis, they do not guarantee 100 % sensitivity or specificity. Therefore, the involvement of other opportunistic pathogens that are not detected by standard diagnostic methods cannot be completely excluded. Further studies and case accumulation are needed to better understand the potential association between JAK inhibitors and opportunistic infections in humans.

## 4 Conclusion

We report the first known case of PJP in a human, associated with the long-term use of oclacitinib, a veterinary JAK inhibitor not approved for human use.

## CRediT authorship contribution statement

**Junko Watanabe:** Writing – review & editing. **Kazuhisa Takahashi:** Writing – review & editing, Supervision. **Daisuke Usuda:** Writing – review & editing, Visualization, Supervision. **Keisuke Oshima:** Writing – original draft, Visualization, Investigation, Data curation, Conceptualization. **Takashi Akimoto:** Writing – review & editing. **Ryo Koyama:** Writing – review & editing, Supervision, Data curation. **Tomohito Takeshige:** Writing – review & editing. **Toshihiko Nishioki:** Writing – review & editing.

## Declaration of Generative AI and AI-assisted technologies in the writing process

The authors declare that no generative AI or AI-assisted technologies were used in the preparation of this article. The entire content was written and reviewed by the authors.

## Informed consent and patient details

Informed consent was obtained from the patient for publication of this case report and accompanying images.

## Funding

The authors received no external funding.

## Declaration of Competing Interest

The authors declare that they have no known competing financial interests or personal relationships that could have appeared to influence the work reported in this paper.

## Data Availability

No datasets were generated or analyzed in this study.
